# Genetic structure of *Mugil cephalus* L. populations from the northern coast of Egypt

**DOI:** 10.14202/vetworld.2016.53-59

**Published:** 2016-01-15

**Authors:** Mahmoud Magdy, Mariam Gergis Eshak, Mohamed Abdel-Salam Rashed

**Affiliations:** 1 Department of Genetics, Faculty of Agriculture, Ain Shams University, 68 Hadayek Shubra, 11241 Cairo, Egypt; 2 Department of Cell Biology, National Research Centre, Dokki, Giza, Egypt

**Keywords:** fluorescent amplified fragment length polymorphism, isolation by distance, Mantel test, marine fish, *Mugil cephalus*, natural selection pressure, population structure

## Abstract

**Aim:**

The gray mullet, *Mugil cephalus*, has been farmed in semi-intensive ponds with tilapia and carps in Egypt for years. The current study used the fluorescent amplified fragment length polymorphism (F-AFLP) technique to search for genetic differences between the populations of *M. cephalus* in the northern region of Egypt and to detect the gene flow between sampled locations and the homogeneity within *M. cephalus* genetic pool in Egypt.

**Materials and Methods:**

To fulfill the study objectives 60 (15/location) samples were collected from four northern coast governorates of Egypt (Alexandria “sea,” Kafr El-Sheikh “farm,” Damietta “farm” and Port Said “sea”). Three replicates of bulked DNA (5 samples/replicate) for each location were successfully amplified using the standard AFLP protocol using fluorescent primers. DNA polymorphism, genetic diversity, and population structure were assessed while positive outlier loci were successfully detected among the sampled locations. Based on the geographical distribution of sampling sites, the gene flow, the genetic differentiation, and correlations to sampling locations were estimated.

**Results:**

A total of 1890 polymorphic bands were scored for all locations, where 765, 1054, 673, and 751 polymorphic bands were scored between samples from Alexandria, Kafr El-Sheikh, Damietta and Port Said, respectively. The effective number of alleles (n_e_) for all bulked samples combined together was 1.42. The expected heterozygosity under Hardy–Weinberg assumption (H_e_) for all bulked samples combined together was 0.28. Bulked samples from Damietta yielded the lowest n_e_ (1.35) and the lowest H_e_ (0.23) when inbreeding coefficient (F_IS_) = 1. Bulked samples from Kafr El-Sheikh scored the highest n_e_ (1.55) and the highest H_e_ (0.37). Bulked samples from Alexandria scored 1.40 for n_e_ and 0.26 for H_e_, while bulked samples from Port Said scored 1.39 for n_e_ and 0.26 for H_e_. The observed bulked samples formed three sub-population groups, where none is limited to a certain sampling location. A high differentiation among locations was detected, however, is not fully isolating the locations. Gene flow was 0.58. Positive outliers loci (117) were detected among the four sampled locations while weak significant correlation (r=0.15, p=0.03) was found for the distance between them.

**Conclusion:**

Even though this species is cultivated in Egypt, the wild population is still present and by the current study a flow of its genes is still exchanged through the northern coast of Egypt. Which contribute to the cultivated populations leading to heterogeneity in its genetic pool and consequently affects the production consistency of *M. cephalus* in Egypt.

## Introduction

The gray mullet, *Mugil cephalus* Linnaeus, is commonly referred to as the striped, gray, or black mullet [1]. The gray mullet has been farmed for centuries in extensive and semi-intensive ponds in many countries. Traditional aquaculture methods employed for raising mullet are now advanced, especially in Italy. Flathead gray mullet is a very important aquaculture species in Egypt, where its farming has been traditional in the “hosha” system in the delta region for centuries. Since the early 1960s, flathead gray mullet has also been cultured in semi-intensive ponds with tilapia and carps in Egypt [2].

*Mugil cephalus* is cosmopolitan in the coastal waters of most tropical and subtropical zones and it is commonly found between 42° N and 42° S [3]. It is catadromous, frequently found coastally in estuaries and freshwater environments. Adult mullet have been found in waters ranging from zero salinity to 75% while juveniles can only tolerate such wide salinity ranges after they reach lengths of 4-7 cm. Flathead gray mullet is a diurnal feeder, consuming mainly zooplankton, dead plant matter, and detritus. Mullet have thick-walled gizzard-like segments in their stomach along with a long gastrointestinal tract that enables them to feed on detritus. Trials on the artificial propagation of flathead gray mullet have been carried out, but most of the commercial aquaculture production of flathead gray mullet still depends on fry collected from the wild, which is cheaper.

Several population genetic studies targeted the gray Mullet habitat in the Mediterranean Sea, Atlantic Ocean and, to a lesser extent, East Pacific and Indian Oceans, as a model of study in order to obtain more information for the biodiversity conservation and fishery management. These studies included allozyme analysis, biochemical markers and mitochondrial DNA sequences [4-12], and more recently, the amplified fragment length polymorphism (AFLP) [13].

The AFLP technique is widely used in phylogenetic and population genomics studies, particularly in non-model organisms for which no prior DNA sequence information is available [14]. Wide multi-locus screening (also known as genome scan) of the locus-specific signature can reflect efficiently the adaptive divergence and genetic differentiation within a population [15]. AFLP-based genome scans have been used successfully to detect genetic differentiation due to adaptation to altitude, adaptation to soil type, insecticide resistance or ecotype divergence [16-22]. The most studies using population genomics approaches conclude that a substantial proportion of the genomes analyzed shows potential signatures of selection (about 5% of the analyzed *loci*; Nosil *et al*. [23]).

The objectives of this study were to use fluorescent AFLP based genome scanning to search for genetic differences within and between the sampled populations of *M. cephalus* and; to detect the gene flow between sampled locations and the homogeneity within *M. cephalus* genetic pool in the northern region of Egypt.

## Materials and Methods

### Ethical approval

The catching of the fish material used in the current study was permitted by the General Authority for Fish Resources Development, Ministry of Agriculture, Egypt and the Animal research ethics approval sub-committee of the Genetics department committee, Faculty of Agriculture, Ain Shams University, Egypt.

### Sampling and DNA extraction

**Figure-1:**
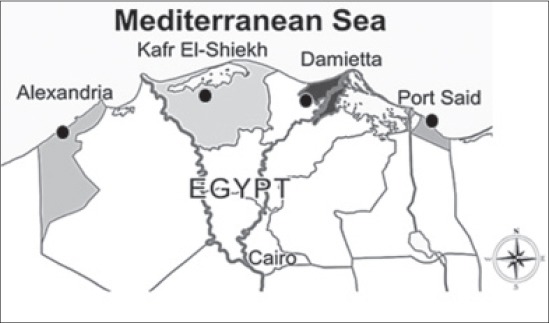
Sampling location along the northern coast governorates of Egypt (Alexandria “sea,” Kafr El-Sheikh “farm,” Damietta “farm” and Port Said “sea”).

Fish samples were gathered from four locations along the northern coast governorates of Egypt (Location 1: Alexandria, sea “latitude: 31.200 and longitude: 29.919,” Location 2: Kafr El-Sheikh, farm “latitude: 31.106 and longitude: 30.942,” Location 3: Damietta, farm “latitude: 30.046 and longitude: 31.254” and Location 4: Port Said, sea “latitude: 31.418 and longitude: 31.814”; Figure-1). The DNA extractions were carried out from fins of 60 collected fish samples (15/location) using Wizard^®^ Genomic DNA Purification Kit (PROMEGA, USA) by following the manufacturer’s manual. DNA quality was then tested using agrose gel electrophoresis (1%) contain 1 µl of ethidium bromide (100 mg/ml), and electrophoresed for 1 h (4V/cm). When successful, DNA was bulked in three replicates (5 samples/replicate), that lead to 12 bulked samples (3-bulked sample/location).

### AFLP-polymerase chain reaction (PCR)

The original protocol of Vos *et al*. [14] was followed using fluorescent primers instead of radioactive agents. All primers and adaptors were synthesized (Invitrogen, UK) and prepared as recommended (Table-1). Six different selective PCR combinations (3 Eco+NNN × 2 Mse+NNN primers) were amplified using the original PCR program. Private Service was contracted to visualize the amplified products using ABI3730 DNA analyzer (Applied Biosystems, USA) with a size standard GS500-LIZ (Macrogen Genescan Service, Korea).

**Table-1:** Sequence 5´- 3´ of primers and adaptors used to establish the AFLP-PCR technique according to the original protocol of Vos *et al.* [14].

Oligo	5´ - sequence - 3´	Oligo	5´ - sequence - 3´
MseI-Adap1	GACGATGAGTCCTGAG	EcoRI-Adap1	CTCGTAGACTGCGTACC
MseI-Adap2	TACTCAGGACTCAT	EcoRI-Adap2	AATTGGTACGCAGTC
Mse-C	GATGAGTCCTGAGTAA**C**	Eco-A	GACTGCGTACCAATTC**A**
Mse-CAA	GATGAGTCCTGAGTAA**CAA**	Eco-ACA	***FAM***-GACTGCGTACCAATTC**ACA**
Mse-CTC	GATGAGTCCTGAGTAA**CTC**	Eco-AGG	***HEX***-GACTGCGTACCAATTC**AGG**
		Eco-ATA	***CY3***-GACTGCGTACCAATTC**ATA**

Selective nucleotide are in bold. AFLP=Amplified fragment length polymorphism, PCR=Polymerase chain reaction

### Data analysis

#### Band scoring

Automated AFLP scoring was performed using two programs Peak Scanner™ (Applied Biosystems, USA) for peak calling and Rawgeno V2 for automated scoring, according to the software’s manuals. The analysis of the AFLP data was based on the band-binary criterion (i.e. codifying the detected bands to, 1 when the presence and 0 when absent) and processed according to Bonin *et al*. [24].

#### Genetic diversity and population structure

To investigate the genetic structure, Bayesian clustering method was applied by using Structure V2.2 [25]. Triple independent simulations were performed per each assumed number of sub-populations K (K=1 to 6). Parameters were set as the following burn-in period of 10,000 out of 100,000 MCMC iterations, and admixture ancestry model was set on.

#### Outlier loci detection

This procedure identifies *loci* which exhibit higher or a lower fixation index (F_ST_) values than the great majority of neutral markers. Mcheza software [26] was used to detect positive outliers considering only polymorphic *loci*. Under the default parameters, Mcheza was run five times with 100,000 simulations at 100% confidence limit. *Loci* that constantly appeared to be an outlier in each run were included in the genetic differentiation analyses.

#### Genetic differentiation, gene flow and geographical influence

Analysis of molecular variance (AMOVA) was performed to test the population genetic differentiation by using Arlequin V3.5 [27]. The significance of F_ST_ was tested with 10000 permutations for the detected AFLP *loci*. Gene flow (Nm) based on F_ST_ value was estimated using AFLP-Surv [28]. The effect of the geographical distance between sampled locations on the distribution of the genotypes of *M. cephalus* was tested using Mantel test (to measure the association between two matrices) implemented in GenAlEx V6 [29]. The genetic distance and log (genetic distance) matrices of AFLP all loci and AFLP positive outlier *loci* were tested against the geographical distance and the log (geographical distance). Data and log data were used to find which were the most appropriate to represent a better correlation using Mantel test [30]. The significance of the correlation value was tested with 10,000 permutations.

## Results

### Fragment analysis and band scoring

**Figure-2:**
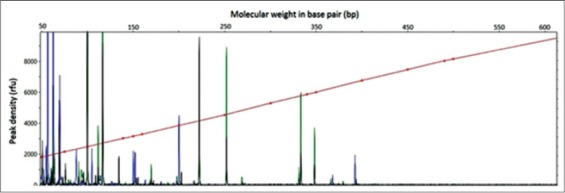
Fragment analysis chromatogram example of bulked sample no. 1 from Alexandria. Multiplexed selective amplified fragment length polymorphism-polymerase chain reaction of: FAM-Eco-ACA × Mse-CAA (blue peaks), HEX-Eco-AGG × Mse-CAA (green peaks) and CY3-Eco-ATA × Mse-CAA (black peaks), are shown. Only peaks within 50-600 bp were considered.

PCR amplification was successful for six pairs of AFLP selective primers. Band scoring for each primer pair gathered bands between 50 and 650 bp (Figure-2). A total of 1890 polymorphic bands were scored from all primer pairs for all the 12 bulked samples. Polymorphic bands for each location were 765, 1054, 673 and 751 for Alexandria, Kafr El-Sheikh, Damietta and Port Said, respectively. The mean band presence was ~799 while the mean fragment size was ~360 bp with a standard deviation of ~160 bp. A weak significant negative correlation was found between fragment sizes and frequencies (r=−0.19; p<0.00).

### Genetic diversity and population structure

The effective number of alleles (n_e_) for all bulked samples combined was 1.42. The expected heterozygosity under Hardy–Weinberg assumption (H_e_) for all bulked samples combined together was 0.28. Bulked samples from Damietta yielded the lowest n_e_ (1.35) and the lowest H_e_ (0.23) when F_IS_=1. Bulked samples from Kafr El-Sheikh scored the highest n_e_ (1.55) and the highest H_e_ (0.37). Bulked samples from Alexandria scored 1.40 for n_e_ and 0.26 for H_e_, while bulked samples from Port Said scored 1.39 for n_e_ and 0.26 for H_e_ (Table-2).

**Table-2:** Genetic diversity and DNA polymorphism based on AFLP bands.

Parameter/location	Alexandria	Kafr El-Sheikh	Damietta	Port said	All samples
Number of polymorphic bands	765	1054	673	751	1890
Mean heterozygosity (H_e_)	0.26	0.37	0.23	0.26	0.28
Standard deviation (H_e_)	0.32	0.33	0.31	0.32	0.32
Mean effective number of alleles (n_e_)	1.40	1.55	1.35	1.39	1.42
Standard deviation (n_e_)	0.49	0.49	0.47	0.48	0.48

AFLP=Amplified fragment length polymorphism

**Figure-3:**
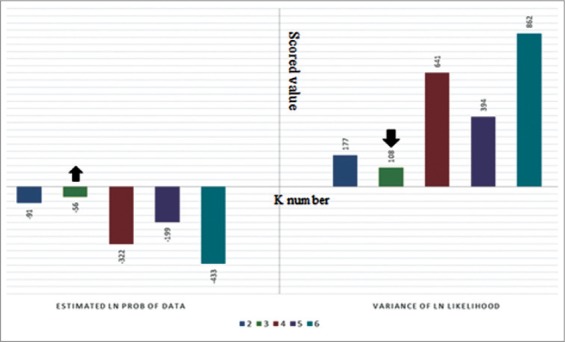
Graphical plotting of structure software output scores based on amplified fragment length polymorphism loci. Estimated ln probability (LnP) and variance of ln likelihood (VLn) for K=[2-6], are shown. K=3 shows the lowest VLn and highest LnP.

The highest average of “estimated Ln probability score” with the lowest variance, sub-population number estimated by the Bayesian inference was K=3, indicating that the observed bulked samples most probably originated from three sub-populations (groups; Figure-3).

**Figure-4:**
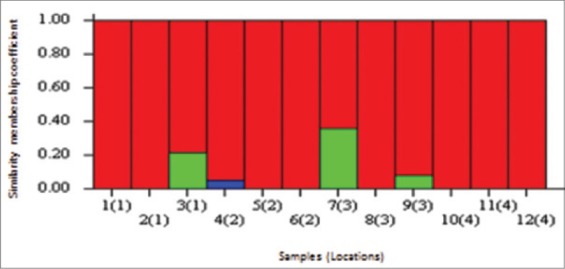
Amplified fragment length polymorphism marker-based structure bar plot graph of K=3, for 12 samples in 4 locations. Samples are ordered by group assignment and locations are indicated between brackets.

Group 1 possesses the highest number of individuals (8) regardless of its geographical location. These are bulked samples number 1 and 2 (Alexandria), 5 and 6 (Kafr El-Sheikh), 8 (Damietta) and 10, 11 and 12 (Port Said). Group 2 consists of a unique bulked sample number 4 from Kafr El-Sheikh location. Group 3 consists of three bulked samples that are number 3 (Alexandria) and 7 and 9 (Damietta). The only homogeneous location that belongs to a certain group was Port Said sampling location (Figure-4).

### Detection of positive selection loci

**Figure-5:**
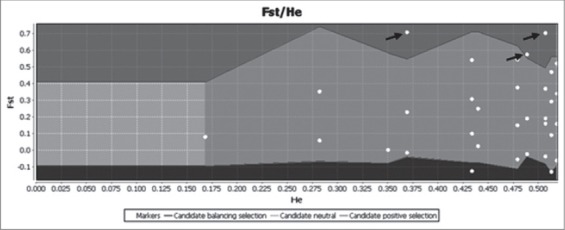
Graphical plot produced by Mcheza software of F_ST_ values against heterozygosity (H_e_) for each of the 1890 amplified fragment length polymorphism (AFLP) loci. The lower and higher zones represent the 0.5% and 99.5% confidence intervals, respectively. Loci in the semi-dark gray zone above the 99.5% are regarded as positive outlier loci. Each dot indicates an AFLP locus, loci scored the same value appears as one dot.

The AFLP data set were analyzed for outlier *loci* detection by using the Mcheza software between the four locations. Across the 16 pairwise analyses between the four groups, 117 out of 1890 polymorphic *loci* (6.19%) were identified as outlier *loci* under directional selection at the 99.5% confidence level (Figure-5). The 117 *loci* appeared constantly as outlier *loci* among the four geographical locations in each run.

### Genetic differentiation, gene flow and geographical influence

An AMOVA test was used to measure the changes in the pairwise differentiation of the *F*_ST_ for the AFLP dataset. F_ST_ of 0.46 (p<0.00), partitioned into a major genetic variation originated within locations, accounting for 53% of the total variations, while 47% of the genetic variation occurred among locations (Table-3). Gene flow (N_m_) was estimated of 0.58 based on Wright’s fixation index (F_ST_=0.46), where “N_m_=[(1/F_ST_)−1]/2.”

**Table-3:** Genetic differentiation through AMOVA of *M. cephalus* based on the AFLP *loci* dataset.

Source of variance	df	SS	Variance components	Percentage of variation
Among locations	3	2395.58	193.09	47
Within locations	8	1754	219.25	53
Total	11	4149.58	412.34	

F_ST_=0.46 (p<0.00). The source of variance (among and within locations), the degree of freedom (df), the SS, the variance components and the percentage of variation, are shown. SS=Sum of squares, AMOVA=Analysis of molecular variance, *M. cephalus=Mugil cephalus*, AFLP=Amplified fragment length polymorphism

Partial Mantel test between the geographical distance and AFLP all loci data set for both data and log data showed no significant correlation, along with the AFLP outlier data against the geographical data. However, weak significant correlation (r=0.15, p=0.03) was found between log (AFLP outlier) matrix against geographical distance matrix.

## Discussion

In Egypt, mullet fish especially *M. cephalus* is economically a very important fish because it has high market value and has been cultivated successfully by fish farmers [31]. Several studies targeted the species *M. cephalus* with many aims, however, less were concerned by its population structure and genetic diversity as it is mainly farmed. Even though, wild populations are still present and by the current study, a flow of its genes are still exchanged through the northern coast of Egypt, thus contribute to the cultivated populations.

The AFLP technique permits a genome-wide scan of the genetic variability with a high number of variable markers. Therefore, there is a relative good chance to detect markers under selection either directly or because they are located near a gene under selection. The high reading output and the extensive statistical refining were expected to reflect more clearly the genetic variability of the studied samples. The mean expected heterozygosity under Hardy–Weinberg assumption (H_e_) was 0.28, which reflects the low diversity level of *M. cephalus* genetic pool from the sampled locations. The structure program implements a model-based clustering method for inferring population structure using molecular data consisting of unlinked markers. The method was introduced by Pritchard *et al*. [32] and extended in sequels by Falush *et al*. [33,34]. The method application is to detect the population structure, identifying distinct genetic groups, assigning samples to sub-populations, and identifying migrants in admixed samples. The bulked samples of Groups 2 and 3 showed mixed portions of Group 1 (inferred by color), which deduce a weak attachment to its assigned cluster, and that they might be grouped to such cluster only when the information about the sampling locations was included. Samples that are genetically related are from different geographical locations, had exactly the same similarity membership coefficient (i.e., a value in which a sample is assigned to a certain group) although they originate from distant locations. Thus, multiple introductions are inferred among sampled locations of *M. cephalus* in the northern coast of Egypt. Such conclusion was further tested and proved by AMOVA.

Currently, there is increasing interest in identifying genes or outlier *loci* that underlie adaptations to different factors in several species [18,35,36]. Outlier *loci* are revealed by unusually high levels of population differentiation at specific marker *loci* [15,37,38]. Those *loci* that are involved in adaptation to local environmental conditions are indeed expected to exhibit increased differentiation among locations along with a decreased diversity within locations [18].

For example, the study of the genetic frame of adaptation to a gradient of altitude in the common frog (*Rana temporaria* L.) by Bonin *et al*. [18] showed that approximately 2% of the AFLP *loci* they screened exhibited elevated altitudinal differentiation. Another recent example was presented by Magdy *et al*. [39] on the cord moss (*Funaria hygrometrica* Hedw.) in which the genome scanning successfully detected loci under selection that were strongly correlated with the gradient of environmental factors in Sierra Nevada mountains. Because local adaptation and directional selection should have *locus*-specific effects of reducing genetic variability within populations and increasing differentiation between populations, *loci* that are outliers for these characteristics are strong candidate regions for involvement in adaptation to the certain environment.

This study is the first report on the detection of candidate *loci* under selection by a genome scan in the *M. cephalus* in the northern coast of Egypt. The AFLP genome scan analysis revealed 117 *loci* as under selection among a total of 1890 *loci* scored in this study. Meyer *et al*. [20] noted that the power of the analysis is directly associated with the genome coverage. These 117 *loci* possess a high credibility because, they were picked up by an exhaustive method (Dfdist embedded in Mcheza software), a very stringent significance criterion of 99.5% was applied, and simulations were set up to the maximum number allowed by the program. Since a high number of reasonable size bands between 150 and 600 bp were found, the *loci* under selection detected here should prove to have a good reliability.

The AMOVA produces estimates of variance components and F-statistics analogs [40]. In our case, AMOVA results were significant; reflect an approximate level of differentiation among sampled locations and within each location (47% and 53%, respectively). Gene flow (N_m_) is a major factor influencing the genetic structure and differentiation among populations. Gene flow was 0.58, the detection of gene flow shows that the genetic differentiation among locations is not absolute. In other words, a complete reproductive isolation between the sampled locations is not the case. Wright [41] proposed that when the gene flow among the locations N_m_>1, the homogenization is the result. When N_m_<1, the locations can be strongly differentiated. According to these criteria, strong genetic differentiation exists among the studied locations.

To elucidate the genetic bases of adaptation to different environments represents a goal of central importance and interest in evolutionary biology [15]. Testing the correlation between the differences in geographic distance among sampled location and the genetic diversity of each sampled location would greatly indicate the geographic influence on the gene flow and further the population structure. Such test is usually known as isolation by distance and was extensively reported during the last decade [42]. The presence of a correlation between the genetic variation and the geographic distance (even though it is weak) along with the detected gene flow between the studied locations, it supports the presence of a certain gene flow limited by the distance between the sampling locations due to some environmental obstacles more than biological reasons. The outliers contribute to such assumption, as it raise the presence of selection mechanism based on the location conditions. However, it is not absolute that wild populations might decrease the isolation level through the gene flow caused by its movement along the northern coast attracted by the zooplankton, dead plant matter, and detritus near the shore, and being used as fries for cultivation [2].

As the detected AFLP *loci* are likely located in non-coding DNA, some of the outlier *loci* may only exhibit the signature of selection because they are linked to the actual target [43]. Although it is difficult to know the location and function of the detected outlier *loci*, the genome scan of *M. cephalus* still offers a window to unravel the genetic basis of fish adaptation without known phenotypes and whole genome sequences. Nowadays, the AFLP primers that were used to amplify the identified outlier *loci* can be used to construct a reduced representation library of the *M. cephalus* genome using next-generation sequencing technology [44].

## Authors’ Contributions

MM has carried out the experimental work in the MAR laboratory and then he prepared the manuscript, while ME has checked the manuscript and polished English language. All authors read and approved the final manuscript.
